# High baseline expression of IL-6 and IL-10 decreased CCR7 B cells in individuals with previous SARS-CoV-2 infection during BNT162b2 vaccination

**DOI:** 10.3389/fimmu.2022.946770

**Published:** 2022-08-16

**Authors:** Alberto Ponciano-Gómez, Martha Iris Valle-Solis, Myriam Campos-Aguilar, Rafael Jijón-Lorenzo, Elena de la C. Herrera-Cogco, Roberto Ramos-Alor, César Isaac Bazán-Mendez, Gustavo Antonio Pérez-Gil Cervantes, Ricardo Ávila-García, Abdiel González Aguilar, Moises Geovani Salmerón Texale, Wilfrido David Tapia-Sánchez, Carlos Leonardo Duarte-Martínez, Sandra Olivas-Quintero, Santiago Cristobal Sigrist-Flores, Itzell Alejandrina Gallardo-Ortíz, Rafael Villalobos-Molina, Adolfo Rene Méndez-Cruz, Rafael Jimenez-Flores, Leopoldo Santos-Argumedo, Juan Pedro Luna-Arias, Hector Romero-Ramírez, Victor Hugo Rosales-García, Bartolo Avendaño-Borromeo

**Affiliations:** ^1^ Laboratorio de Inmunología, Unidad de Morfología y Función, Facultad de Estudios Superiores Iztacala, Universidad Nacional Autónoma de México, Tlalnepantla, Estado de México, Mexico; ^2^ Secretaría de Salud de Veracruz, Servicios de Salud de Veracruz, SESVER, Xalapa Veracruz, Mexico; ^3^ Laboratorio Estatal de Salud Pública, Gobierno del Estado de Veracruz, Veracruz, Mexico; ^4^ Laboratorio de Citometría de Flujo y Hematología, Diagnóstico Molecular de Leucemias y Terapia Celular (DILETEC), Gustavo A. Madero, Ciudad de Mexico, Mexico; ^5^ Department of Health Sciences, Autonomus University of Occident, Culiacan, Sinaloa, Mexico; ^6^ Unidad de Biomedicina, Facultad de Estudios Superiores Iztacala, Universidad Nacional Autónoma de México, Tlalnepantla, Estado de México, Mexico; ^7^ Departamento de Biomedicina Molecular, Centro de Investigación y de Estudios Avanzados del Instituto Politécnico Nacional (CINVESTAV-IPN), Ciudad de México, Mexico; ^8^ Departamento de Biología Celular, Centro de Investigación y de Estudios Avanzados del Instituto Politécnico Nacional (CINVESTAV-IPN), Ciudad de México, Mexico; ^9^ Laboratorios Nacionales de Servicios Experimentales, Centro de Investigación y de Estudios Avanzados del Instituto Politécnico Nacional, Ciudad de México, Mexico

**Keywords:** SARS-CoV-2, BNT162b2, Interleukin 6, Interleukin 10, CCR7 B cells

## Abstract

The current pandemic generated by SARS-CoV-2 has led to mass vaccination with different biologics that have shown wide variations among human populations according to the origin and formulation of the vaccine. Studies evaluating the response in individuals with a natural infection before vaccination have been limited to antibody titer analysis and evaluating a few humoral and cellular response markers, showing a more rapid and intense humoral response than individuals without prior infection. However, the basis of these differences has not been explored in depth. In the present work, we analyzed a group of pro and anti-inflammatory cytokines, antibody titers, and cell populations in peripheral blood of individuals with previous SARS-CoV-2 infection using BNT162b2 biologic. Our results suggest that higher antibody concentration in individuals with an earlier disease could be generated by higher production of plasma cells to the detriment of the presence of memory B cells in the bloodstream, which could be related to the high baseline expression of cytokines (IL-6 and IL-10) before vaccination.

## Introduction

The SARS-CoV-2 infection has revealed gaps in the immune response concerning coronaviruses affecting human populations (1–3). Many publications have evaluated the antibody production in infected and vaccinated people (4–6). Antibody levels vary from very low in patients with mild or asymptomatic infections to high levels in hospitalized infected patients (7–10). The massive vaccination with different biologics has also shown wide variations among human populations depending on the origin and vaccine formulation (5, 6, 11). However, there are scarce studies where antibody titers have been measured by comparing healthy people with vaccinated people who have suffered a SARS-CoV-2 infection (12–14). These studies have shown that a previous infection correlates with higher antibody titers. However, the bases for these differences have not been explored in depth.

This work determined the antibody responses in a sample of people vaccinated with the biological BNT162b2, separating the population into people who previously suffered or did not have an infection with SARS-CoV-2. In addition to the antibody titers, various cell populations and pro- and anti-inflammatory cytokines were analyzed. Given the number of parameters studied in this work, we decided to use a principal component analysis (PCA), looking for those parameters that could best correlate with the differences in antibodies that PCA allowed by concentrating on a few parameters, simplifying the analysis.

The population of B lymphocytes expressing the chemokine receptor CCR7 decreased in those who previously had an infection with SARS-CoV-2. Likewise, people showed increased IL-10, IL-12p70, and IL-6 levels once infected. Interleukins 6 and 10 participate in the differentiation of activated B lymphocytes towards plasma cells, which could correlate with higher antibody titers. In contrast, IL-12p70 could participate, *via* gamma interferon stimulation, in the change of isotype towards IgG.

Our results suggest that people who suffered a previous SARS-CoV-2 infection once vaccinated with the biological BNT162b2 generate a more significant production of plasma cells to the detriment of the generation of memory B lymphocytes circulating through the secondary lymph nodes.

## Materials and methods

### Study population and sampling

This work was a longitudinal observational study in a single health center, including adults with previous SARS-CoV-2 infection and naïve. The local Ethical Committee approved the study (CE/FESI/022021/1380). A total of 65 individuals from the staff of the Health Institutes of Veracruz, Mexico, vaccinated with the Pfizer-BioNTech biological (BNT162b2) were selected 20 with a previous SARS-CoV-2 infection (pre-infected) and 45 naïve (**Table 1**). The peripheral blood samples for the analysis were taken on day 0, before the first dose (T1), the day of the second dose (T2), and the third sample was obtained 14 days after the boost (T3).

**Table 1:** General data of the study population.

	Naïve	Pre-infected
Number of individuals	45	20
Women(%)	67	65
Men (%)	33	35
average age (years)	42.2 (12)	39.9 (13)
minimum age (years)	24	29
maximum age(years)	57	58
Median age (years)	44	39
1st Quartile age (years)	37	36
3rd Quartile age (years)	49	49
Mean time from the onset of infection to the first dose (months)	NA	6.6 (6)
Minimum time from onset of infection to the first dose (months)	NA	1
Maximum time from onset of infection to the first dose (months)	NA	10
Median time from onset of infection to the first dose (months)	NA	7
1st Quartile time from onset of infection to the first dose (months)	NA	3
3rd Quartile time from onset of infection to the first dose (months)	NA	9

The study population is described, including the mean and, in parentheses, the ICR of the quantitative variables.

NA, not applicable.

### Cytokine quantification by flow cytometry

The panel of cytokines IL-1β, IL-2, IL-4, IL-6, IL-10, IL-12p70, IL-17A, CXCL8 (IL-8), CXCL10 (IP-10), CCL2 (MCP-1), IFN-γ, TNF-α, and TGF-β1 were measured in the peripheral blood plasma using LEGENDplex™ HU Essential Immune Response Panel (13-plex) kits (Biolegend), according to the manufacturer’s instructions to quantify the absolute values of cytokines. The flow cytometry data acquisition was performed with (CytoFLEX S, Beckman Coulter) equipment, and the results were analyzed using the LEGENDplex™ software.

### Cell population quantification by flow cytometry

Multicolor staining with monoclonal antibodies and flow cytometry was used to identify subpopulations of B lymphocytes (CD19+, CD20+, CD19+ CD27+, CD20+ CD27+, CD19+ CCR7+, CD20+ CCR7+), T lymphocytes (CD3+ CD4+, CD3+ CD8+,CD4+ CD25+ CD127Low, CD3+ CD4+ CD45Ra+, CD3+ CD4+ CD45Ra+ CCR7+, CD3+ CD8+ CD45Ra+, CD3+ CD8+ CD45Ra+ CCR7+), NK lymphocytes (CD56+, CD57+, CD3- CD16+ CD56+, CD3- CD16+ CD57+) and monocytes (CD14+, CD14+ CCR7+, CD14+ TLR4+, CD14+ TLR4+ CCR7+ CD11c+, HLA-DR+) (**Supplementary Figure 1**). A volume of whole blood with 1 × 10^6^ white blood cells (previously counted with hemocytometer) were stained with the four antibody panels and incubated for 30 minutes at room temperature in darkness, washed with phosphate-buffered saline containing 0.1% bovine serum albumin, and lysed with the OptiLyse C reagent (Beckman Coulter) following the manufacturer’s recommendations. Samples were analyzed with the Cytoflex S system (Beckman Coulter), 100,000 events acquired in each of the four panels used, and data was analyzed with the Kaluza C software (Beckman Coulter).

### Anti-SARS-CoV-2 quantification by flow cytometry

The anti-SARS-CoV-2 anti-spike and anti-RBD antibodies were quantified in plasma using the LEGENDplex™ SARS-CoV-2 Serological IgG Panel Detection Abs (Biolegend), following the manufacturer’s instructions for determining the absolute antibody values. The acquisition was carried out by flow cytometry (Cytoflex S), and the results were analyzed with the LEGENDplex software.

### Principal component analysis (PCA)

Through the FactoMineR package in R software, we performed a PCA, which summarizes and visualizes the information in all our data sets to describe multiple inter-correlated quantitative variables; we also added a concentration ellipse around pre-infected and naïve clusters from a mean point using the default confidence level (0.95) underlying Gaussian distribution. We used PCA to extract the essential variables to express the principal components (variables) involved in differentiating the response to the BNT162b2 vaccine between pre-infected and naïve individuals.

### Statistical analysis

After the PCA analysis, we selected the normalized group data, compared the most representative variables between the different clusters, and did a Student’s t-test. Values with a confidence interval of 95 and P-values ≤ 0.05 were considered statistically significant.

## Results

### Antibodies production

**Figure 1:**
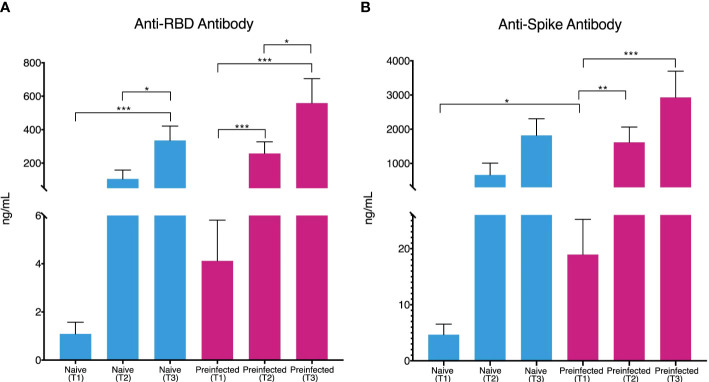
Production of Anti-RBD and Anti-Spike antibodies during vaccination. Individuals without previous infection (blue bars, n= 45) and with previous SARS-CoV-2 infection (red bars, n = 20) showed an increase in the concentration (ng/mL) of Anti-RBD antibodies **(A)** throughout the three times evaluated [T1 (1.084 and 4.12 mean), T2 (105.32 and 258.11 mean) and T3 (334.91 and 559.03 mean)]. Anti-Spike antibody concentration **(B)** showed the same behavior [T1 (4.65 and 18.94 mean), T2 (664.71 and 1615.25 mean), and T3 (1819.72 and 2930.08 mean)]. (*p ≤ 0.05, **p ≤ 0.01, ***p ≤ 0.001, two-sided t-test). vertical lines show the standard error.

Anti-RBD and anti-Spike antibodies were determined at the three-time points, T1, T2, and T3. In pre-infected SARS-CoV-2 individuals, anti-RBD (4.32 ng/mL on average) and anti-Spike (19.84 ng/mL antibodies were identified on average from the T1 moment of the first vaccination (**Figures 1A, B**). In the case of pre-infected and naive individuals, the concentration of anti-Spike and anti-RBD antibodies increased steadily during follow-up, reaching an average concentration of 2930 ng/mL and 559.03 ng/mL, respectively at the last sampling. In the case of the anti-Spike antibodies of the pre-infected individuals, 2930 ng/mL and 2325.1 ng/µl for the naïve individuals (**Figure 1B**); while the average values reached for the anti-RBD antibodies were 334.921 ng/µL for the naive individuals and 559.03 ng/mL in the case of pre-infected individuals (**Figure 1A**).

### Principal component analysis (PCA)

**Figure 2:**
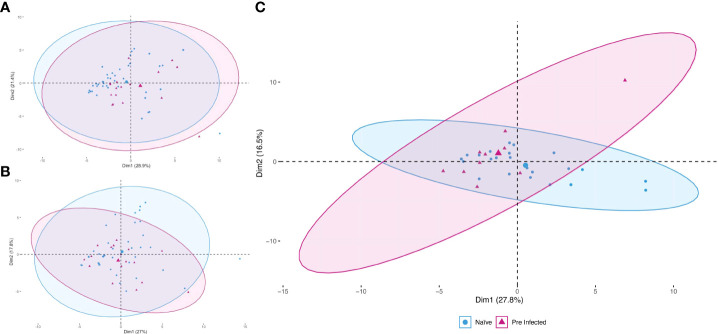
Principal component analysis during vaccination. The study was applied during the three times evaluated [T1 **(A)**, T2 **(B)**, and T3 **(C)**]. Red triangles represent pre-infected individuals (n = 20). Blue circles represent individuals without prior SARS-CoV-2 infection (n = 45). The more prominent symbols represent each population’s centroid (mean), and the concentration ellipses represent the estimates according to a Gaussian distribution at a 95% confidence level for each group.

To reduce the dimensionality of the multivariate and to address the complexity of the immune response generated by the vaccine in the population studied with minimal loss of information, we constructed a PCA at each of the three moments (T1, T2, and T3), using values of all the evaluated subpopulations cells and the determination of cytokines (**Figure 2**). Considering that the size of the constructed ellipse depends directly on the variance between the data, we can assume that the differences in cell subpopulations and cytokine concentration between pre-infected and naive clusters at times T1 and T2 (**Figures 2A, B**) are less than the variation that occurs between these two groups in T3 (**Figure 2C**).

### Modified cell populations

**Figure 3:**
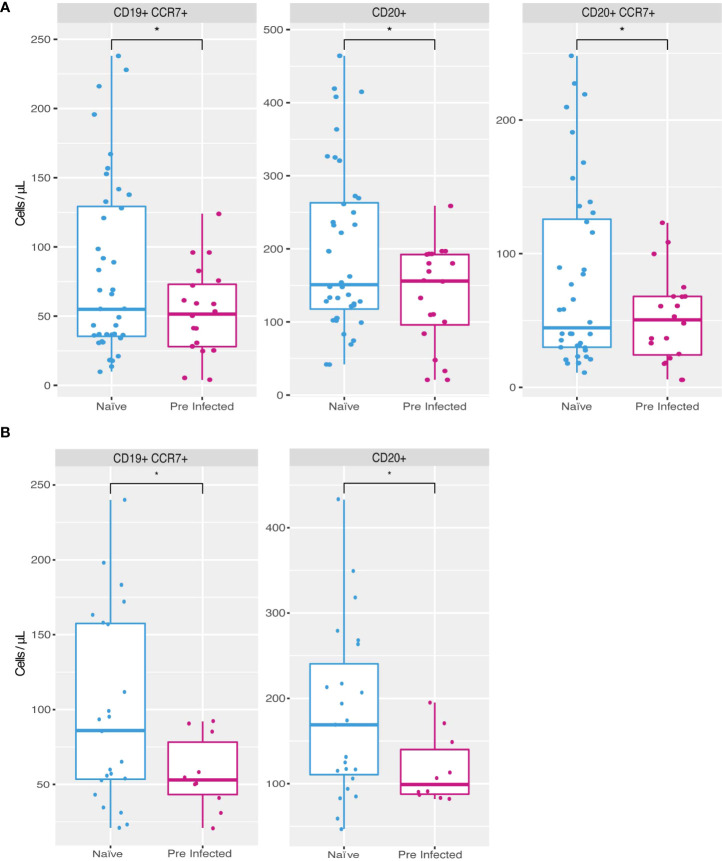
Increased cell populations in individuals without previous SARS-CoV-2 infection. Naive individuals (n = 45) showed higher counts of CD19+ CCR7+ (81.9 cells/µl mean), CD20+ (196.87 cells/µl mean) and CD20+ CCR7+ (79.95 cells/µl mean) cells during time 2 **(A)** compared to previously infected individuals (n = 20) (52.85, 136.6 and 51.8 cells/µl mean respectively). During time 3 **(B)** naive individuals presented a higher count of CD19+ CCR7+ (98 cells/µl on average) and CD20+ (181.04 cells/µl mean) populations compared to previously infected individuals (57.5 and 116.8 cells/µl mean, respectively) (*p ≤ 0.05, two-sided t-test).

The PCA allowed us to summarize and identify the most critical parameters that differentiate the response generated by the vaccine in pre-infection compared with naive individuals. Of these principal components, the cell populations that showed statistically significant differences are B lymphocytes, specifically the populations of CD19+ CCR7+, CD20+ and CD20+ CCR7+ cells (**Figure 3A**). In addition, all these populations were reduced in individuals with a previous infection compared to naive individuals. Both results were observed at the second vaccine dose (T2) and on day 14 after the boost (T3) (**Figures 3A, B**), meanwhile on day zero, before the application of the first dose (T1), no significant difference was observed in these cell markers (data not shown), this could be due to the variation in the time of infection and vaccination in the population studied (**Table 1**).

### Modified cytokines

**Figure 4:**
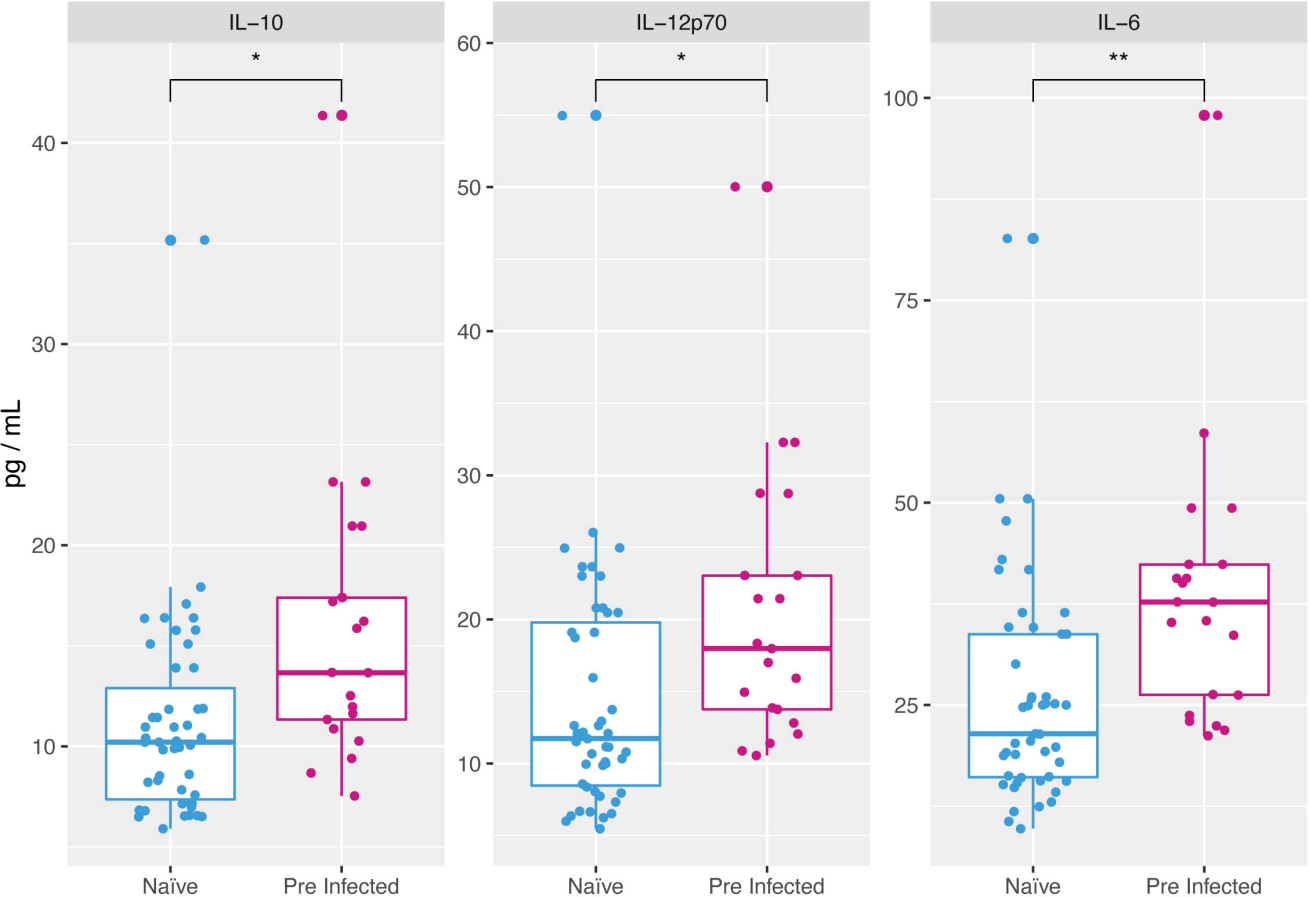
Cytokines overexpressed at the beginning of vaccination in individuals with previous SARS-CoV-2 infection. At time 1 naive individuals presented a lower concentration of cytokines IL-10 (11.08 pg/ml mean), IL-12p70 (14.4 pg/ml mean) and IL-6 (25.94 pg/ml mean) compared to individuals with pre-vaccination SARS-CoV-2 infection (15.96, 20.50, and 38.36 cells/µl mean respectively) (*p ≤ 0.05, **p ≤ 0.01, two-sided t-test).

Of the principal components obtained from PCA that correspond to cytokines, IL-10 (32.5 pg/mL from naïve and 29.3 pg/mL from pre-infected average concentration), IL-12p70 (28.9 pg/mL from naïve and 29.4 pg/mL from pre-infected average concentration), and IL-6 (62.5 pg/mL from naïve and 64.0 pg/mL from pre-infected average concentration) showed significant differences (p ≤ 0.005) (**Figure 4**), the concentration is higher in individuals with an infection before vaccination. These differences are observable only before administering the vaccine’s first dose.

## Discussion

Consistently, with prior studies, we have found that individuals with a previous infection show anti-Spike and anti-RBD antibody titers before administering the first dose of the vaccine, unlike individuals without the previous disease who showed lower values (14, 15). Individuals in the first group (previous infection) tested positive for SARS-CoV-2 before the vaccine and generated a humoral immune response. After the first dose, antibody production is higher in individuals with previous immunity (**Figure 1**), consistent with findings suggesting that a seropositive state causes a more rapid antibody response to vaccination and reinforces the justification for considering a one-dose vaccine regimen in this population (14, 15).

Our monitoring of antibody titers throughout the vaccination process (including the second dose and 14 days later) showed that the response in individuals with immunity before vaccination is more remarkable, as reported by previous work (13, 16). In addition, individuals with prior infection showed anti-Spike and anti-RBD antibody titers two-fold higher than individuals without disease before vaccination (**Figure 1**), suggesting that immunity before immunization led to a more intense response, not only after the first dose but throughout the entire vaccine-induced response, providing more robust and longer-lasting protection against infection (17).

Although this more intense response in the production of antibodies has been previously reported by (14, 15, 18, 19), it is clear that there may be more differences in the immune response mounted by individuals with natural infection and without previous natural disease, and some of them could even help to explain the disparity in the humoral response. We evaluated a panel of cytokines and cell populations in peripheral blood and the follow-up to identify these differences. These data were analyzed using a PCA which allowed us to explore and reduce this large set of data, increasing the interpretability and minimizing the loss of information; showing that the study groups begin to show variance from T2, increasing in T3 (**Figure 2**), a result that coincides with reports where the immune response due to the immunization process becomes evident from day 14 (20).

The resulting principal components and their pattern throughout the follow-up differed only by a couple of elements between the two working groups; however, a higher concentration of pre-vaccination of IL-6 IL-10 and IL12p70 in naturally infected individuals (**Figure 4**). Although these cytokines have not been previously analyzed in response to vaccination, recent work has shown that these cytokines increased their concentration very early during COVID-19 and that their function could be related to the severity of the SARS-CoV-2 infection (21–24).

In the case of IL-6, it is thought that this pro-inflammatory cytokine could be part of an innate inflammatory response that precedes an adaptive response in natural infection, including SARS-CoV-2 infection (24, 25). Thus, the basal concentration is higher in individuals with the previous disease (**Figure 4**), i.e., it could be related to an earlier induction of an adaptive response, promoting a rapid humoral reaction mediated by the differentiation and proliferation of B cells (24), and therefore with the higher concentration of antibodies in these same individuals. To analyze this possible correlation, we performed a linear regression analysis between IL-6 production in pre-infected individuals and antibody production, finding a Correlation Coefficient with an R value lower than 0.5 (Anti-RBD/IL-6 R = 0.006 and Anti-Spike/IL-6 R = 0.003). Therefore, there is no direct correlation between I-L6 concentration, and the antibody titer found, which is evidence that the increase of IL-6 in the pre-infected individuals may be participating in the increase of the antibody production seen. However, they do not seem to be the only signals responsible for this process. Some other cytokines, receptors, and signaling pathways must be involved.

IL-10 has recently been reported as a crucial biomarker of severity and mortality in patients with COVID-19 disease (21, 26). The early expression of IL-10 could have an anti-inflammatory or immunosuppressive effect, preventing the hyper inflammation that characterizes SARS-CoV-2 infection (24). However, it has been reported that when secreted by regulatory T lymphocytes in patients with severe COVID-19 disease, it would decrease the immune response mediated by T lymphocytes and even their depletion in peripheral blood (27–30). Thus, in the current case of vaccinated individuals with previous infection and high basal levels of IL-10 (**Figure 4**), this cytokine could decrease the immune response mediated by T lymphocytes to the vaccine but, on the other hand, polarized it to a strong response mediated by B lymphocytes.

Although other studies have shown a differential effect at the serological level of vaccination in individuals with a previous infection by SARS-CoV-2 (13–16), few studies have addressed the differences *in vivo* in the quantification of leukocyte populations in peripheral blood.

*In vitro* evidence of a response mediated by T lymphocytes in individuals with previous infection and the application of a single dose of BNT162b2 is absent or minimal in individuals without previous disease (31, 32). Our analysis considered the identification of different subpopulations of T lymphocytes. However, no significant differences were observed between the two study groups throughout the follow-up. This result is possibly due to the need to look for other subpopulations of T cells, which may be analyzed by future research.

In the case of other cell populations, our work did identify modifications in B lymphocyte populations in individuals with a previous infection to vaccination, specifically a decrease in the count of B lymphocytes in peripheral blood that express the chemokine receptor CCR7 (**Figure 3**).

The expression of CCR7 has been previously reported in mature B cells on the way to differentiate into antibody-secreting plasmablasts (33, 34). Therefore, the lower count of these cells in peripheral blood could be related to their migration to the secondary lymphoid organs. They differentiate into plasmablasts and thus increase the concentration of antibodies, which coincides with our finding of a higher average concentration of individuals with the previous infection. However, monitoring these memory plasma cells in peripheral lymphoid organs is almost impossible due to the difficulty of obtaining lymphoid samples from voluntary individuals. For this reason, it could be confirmed using model organisms in future works.

Our work is not the first to report a differential response of B cells from individuals with previous infection and the use of the vaccine (4, 12). However, it does coincide with reports where infection induced a modification in the production of antibodies. This result is possibly related to the activation of memory B lymphocytes (12), which, as our findings suggest, could be generated at the expense of a decrease in circulating mature B lymphocytes (**Figure 3**).

More importantly, this increased immune response in previously infected individuals could be related to the higher baseline of specific cytokines before vaccination. An analysis with a broader panel of cytokines, including those reported here that participate in the natural response to SARS-CoV-2 infection (24), might reveal a mechanism to explain the higher response and possibly more protective in those suffering SARS-CoV-2 infection before the use of the vaccine.

## Data availability statement

The raw data supporting the conclusions of this article will be made available by the authors, without undue reservation.

## Ethics statement

The work was approved by the local ethics committee under registration number (CE/FESI/022021/1380). The participants provided their written informed consent to participate in this study.

## Author contributions

AP-G: First authorship. MV: Equal contribution and first authorship. MC-A, RJ, EH, RR, CB, GP-G, RÁ, AG, MS, WT-S, CD-M, SO-Q, SS-F, IG-O, RV-M, AM-C, RJ-F, LS-A, JL, HR-R, and VR-G: Equal contribution. BA-B: Senior authorship. All authors contributed to the article and approved the submitted version.

## Funding

This research was supported by the Programa de Apoyo a Proyectos de Investigación e Innovación Tecnológica (PAPIIT) grant of Direccion General de Asuntos del Personal Academico (DGAPA), IA209620, and Secretaría de Salud y Servicios de Salud de Veracruz.

## Acknowledgments

We thank Ingineer Cuitláhuac García Jiménez, Constitutional Governor of the State of Veracruz de Ignacio de la Llave, who in his integrative and transformational vision has given an unprecedented boost, taking advantage of the experience and knowledge of human capital, to contribute to the development of the people of Veracruz in terms of health. We would like to thank Dr. Gerardo Dìaz-Morales for his support in the development of this project.

## Conflict of interest

The authors declare that the research was conducted in the absence of any commercial or financial relationships that could be construed as a potential conflict of interest.

## Publisher’s note

All claims expressed in this article are solely those of the authors and do not necessarily represent those of their affiliated organizations, or those of the publisher, the editors and the reviewers. Any product that may be evaluated in this article, or claim that may be made by its manufacturer, is not guaranteed or endorsed by the publisher.
