# Supplementary material for: High baseline expression of IL-6 and IL-10 decreased CCR7 B cells in individuals with previous SARS-CoV-2 infection during BNT162b2 vaccination
Source: Front Immunol. 2022 Aug 16;13:946770. doi: 10.3389/fimmu.2022.946770 (PMC9425053; doi:10.3389/fimmu.2022.946770)
Supplement: Supplementary file 1 [file DataSheet_1.docx]

**Supplementary Material**

**
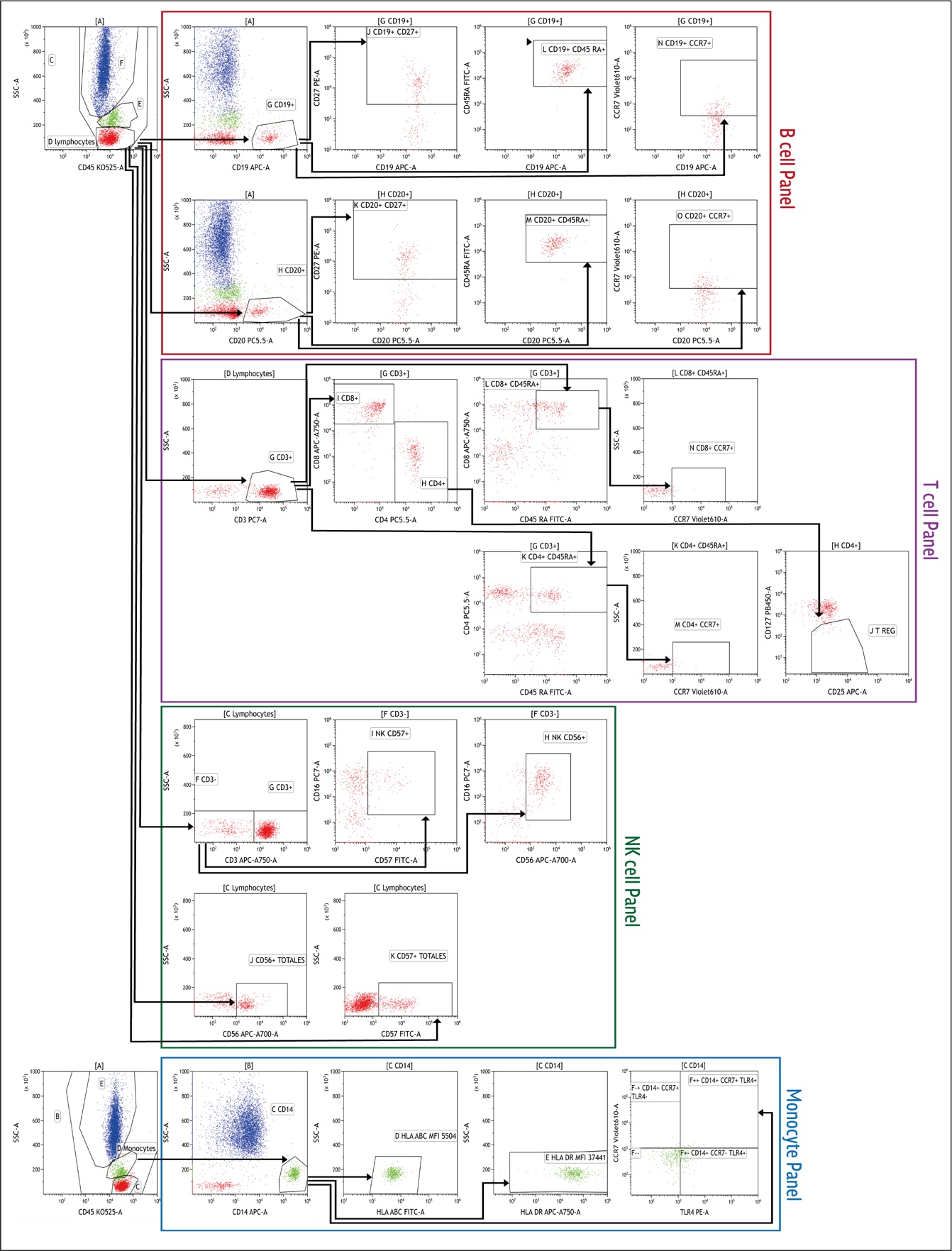
**

**Supplementary Figure 1. Gating strategy.** Stepwise for identifcation of B cell (red section), T cell (violet section), NK cell (Green section), and Monocyte populations (blue section).
